# Curcumin enhances the apoptosis-inducing potential of TRAIL in prostate cancer cells: molecular mechanisms of apoptosis, migration and angiogenesis

**DOI:** 10.1186/1750-2187-2-10

**Published:** 2007-10-04

**Authors:** Sharmila Shankar, Qinghe Chen, Krishna Sarva, Imtiaz Siddiqui, Rakesh K Srivastava

**Affiliations:** 1 Department of Biochemistry, University of Texas Health Science Center at Tyler, Tyler, Texas, 75703, USA

## Abstract

**Background:**

We have recently shown that curcumin (a diferuloylmethane) inhibits growth and induces apoptosis, and also demonstrated that TRAIL induces apoptosis by binding to specific cell surface death receptors in prostate cancer cells. The objectives of this paper were to investigate the molecular mechanisms by which curcumin enhanced the apoptosis-inducing potential of TRAIL in prostate cancer cells.

**Results:**

Curcumin enhanced the apoptosis-inducing potential of TRAIL in androgen-unresponsive PC-3 cells and sensitized androgen-responsive TRAIL-resistant LNCaP cells. Curcumin inhibited the expressions of Bcl-2, Bcl-X_L_, survivin and XIAP, and induced the expressions Bax, Bak, PUMA, Bim, and Noxa and death receptors (TRAIL-R1/DR4 and TRAIL-R2/DR5) in both cell lines. Overexpression of dominant negative FADD inhibited the interactive effects of curcumin and TRAIL on apoptosis. Treatment of these cells with curcumin resulted in activation of caspase-3, and caspase-9, and drop in mitochondrial membrane potential, and these events were further enhanced when combined with TRAIL. Curcumin inhibited capillary tube formation and migration of HUVEC cells and these effects were further enhanced in the presence of MEK1/2 inhibitor PD98059.

**Conclusion:**

The ability of curcumin to inhibit capillary tube formation and cell migration, and enhance the therapeutic potential of TRAIL suggests that curcumin alone or in combination with TRAIL can be used for prostate cancer prevention and/or therapy.

## Background

Prostate cancer is the second largest incidence among the male populations in the US, and the incidence has been increasing rapidly in the recent years [1]. Chemotherapy has provided significant survival benefit in the treatment of prostate cancer; however, it is associated with significant normal tissue toxicity, highlighting the need for therapeutic strategies that target tumor cells without compromising normal tissue function [2]. Increased concentrations of cytotoxic drugs and higher dosages of irradiation often fail to improve the health of prostate cancer patients, and may cause resistance to apoptosis. Thus, it is imperative to identify anticancer agents that are nontoxic and highly effective in inducing apoptosis preferentially in tumor cells.

Epidemiological data support the concept that naturally occurring compounds in the human diet are safe, non-toxic, and have long lasting beneficial effects on human health. Curcumin [1,7-bis(4-hydroxy-3-methoxyphenyl)-1,6-hepatadiene-3,5-dione; diferulolylmethane], a major constituent of the yellow spice turmeric derived from the rhizomes of *Curcuma spp., *is one such compound [3]. It has been used in Asian food for centuries [3]. Curcumin has been reported to have several pharmacological effects including anti-tumor, anti-inflammatory and anti-oxidant properties [3-7]. Recent studies have also suggested that it can inhibit tumor metastasis, invasion and angiogenesis [7-11]. We have recently shown that curcumin induces apoptosis in prostate cancer cells by inhibiting Akt activity upstream of mitochondria, and Bax and Bak genes completely inhibit curcumin-induced apoptosis [12,13]. Furthermore, curcumin inhibits NFκB activity in cancer cells [8,14] and sensitizes cancer cells to chemotherapy and radiotherapy [15-21].

Binding of TRAIL to its receptors TRAIL-R1/DR4 and TRAIL-R2/DR5, both of which contain a cytoplasmic region of 80 amino acids designated as the "death domain", activated the extrinsic apoptosis pathway. Death receptors DR4 and DR5 can recruit the initiator caspases, caspase-8 and caspase-10, by a homotypic interaction between the death effector domains of the adapter molecule Fas-associated death domain (FADD) protein and the prodomain of the initiator caspase, thereby forming the death-inducing signaling complex (DISC). The formation of active DISC is essential for TRAIL to transmit apoptotic signals. We and others have shown that tumor-selective targeting molecules such as tumor necrosis factor (TNF)-related apoptosis-inducing ligand (TRAIL) induces apoptosis in prostate cancer cells, both *in vitro *and *in vivo *[22-25]. Data on experimental animals and primates led us to believe that TRAIL has great promise as a selective anticancer agent [22,23,26]. We have recently demonstrated that TRAIL induces apoptosis in several prostate cancer cells lines, but it was ineffective in inducing apoptosis in LNCaP cells [22,23,27]. Chemopreventive agent curcumin has been shown to sensitize TRAIL-resistant prostate cancer cells *in vitro *[28-30]. However, the molecular mechanisms by which curcumin sensitizes prostate cancer cells to TRAIL treatment are not well understood.

Angiogenesis, the formation of new blood vessels from preexisting capillaries, is essential for tumor progression and metastasis, and consists of a multistep process involving an array of molecular signals [31,32]. During tumor neovascularization, multiple processes (including the stimulation of endothelial cell proliferation, migration and assembly; the recruitment of perivascular cells; and extracellular matrix modeling) are involved. Ras-Raf-MEK-ERK signal transduction pathway has been shown to play an active role in angiogenesis. It is not clear whether ERK plays an active role in antiangiogenic effects of curcumin.

The purpose of our studies was to investigate the molecular mechanisms by which curcumin enhanced therapeutic potential of TRAIL in prostate cancer cells. Our results indicated that curcumin enhanced apoptosis-inducing potential of TRAIL in androgen-unresponsive PC-3 cells and sensitized androgen-responsive TRAIL-resistant LNCaP cells. Curcumin also inhibited capillary tube formation and migration of HUVEC cells and these effects were further enhanced in the presence of MEK inhibitor. Thus, curcumin can be combined with TRAIL to kill androgen-responsive and – unresponsive prostate cancer cells.

## Results

### Curcumin enhances the apoptosis-inducing potential of TRAIL in PC-3 cells and sensitizes TRAIL-resistant LNCaP cells

**Figure 1:**
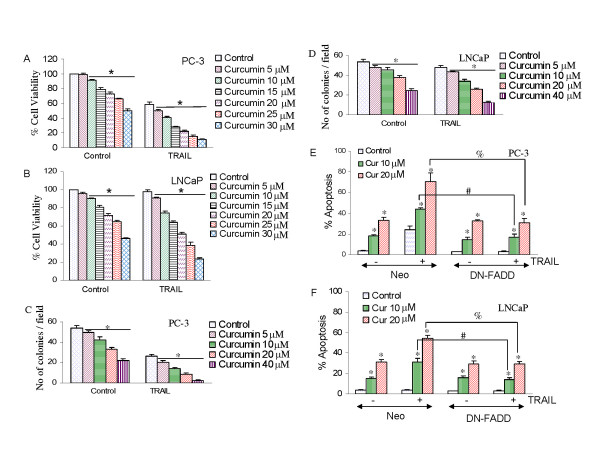
**Interactive effects of curcumin and TRAIL on cell viability and colony formation of prostate cancer cells**. (A), PC-3 cells were treated with various concentrations of curcumin (0–30 μM) for 24 h, followed by treatment with TRAIL (25 nM) for another 24 h. Cell viability was measured at the end of 48 h by XTT assay. (B), LNCaP cells were treated with various concentrations of curcumin (0–30 μM) for 24 h, followed by treatment with TRAIL (50 nM) for another 24 h. Cell viability was measured at the end of 48 h by XTT assay. (C), PC-3 cells were seeded in soft agar and treated with curcumin (5–40 μM) in the presence or absence of TRAIL (25 nM). After three weeks, no of colonies were counted. Data represent mean ± SE. (D), LNCaP cells were seeded in soft agar and treated with curcumin (5–40 μM) in the presence or absence of TRAIL (50 nM). After three weeks, no of colonies were counted. Data represent mean ± SE. (E and F), Effects of dominant negative FADD on curcumin and/or TRAIL-induced apoptosis. PC-3 and LNCaP cells were transiently transfected with either control plasmid or plasmid expressing dominant negative FADD (DN-FADD) along with plasmid (pCMV-LacZ) encoding the β-galactosidase (β-Gal) enzyme. There was no difference in transfection efficiency among groups. Transfected cells were treated with curcumin (0, 10 or 20 μM) in the presence or absence of TRAIL (25 nM for PC-3 cells or 50 nM for LNCaP) for 48 h. Apoptosis was measured by DAPI staining. Data represent mean ± SE. * = significantly different from respective control; # and % = treatment groups were significantly different, P < 0.05.

We first measured the effects of curcumin, with or without TRAIL, on cell viability by XTT assay (Fig. 1). Curcumin inhibited cell viability in prostate cancer PC-3 and LNCaP cells in a dose-dependent manner. TRAIL inhibited cell viability in PC-3 cells (Fig. 1A) but had no effect in LNCaP cells (Fig. 1B). Furthermore, curcumin enhanced the inhibitory effects of TRAIL on cell viability in PC-3 cells, and sensitized TRAIL-resistant LNCaP cells. These data suggest that curcumin alone is effective in both PC-3 and LNCaP cells, enhances the anti-proliferative activity of TRAIL in PC-3 cells, and sensitizes TRAIL-resistant LNCaP cells.

Anchorage independent colony growth is one of the characteristics of cancer cells. We next measured the interactive effects of curcumin and TRAIL on colony growth in soft agar (Fig. 1C and 1D). Curcumin inhibited number of PC-3 and LNCaP colonies formed in soft agar. Colonies formed by PC-3 cells were more sensitive to curcumin than those formed by LNCaP cells. By combination, TRAIL inhibited number of PC-3 colonies, but had no effect on LNCaP colonies. Interestingly, curcumin enhanced the inhibitory effects of TRAIL on PC-3 cell colony formation, and sensitized LNCaP cell colonies. These data suggest that curcumin has ability to inhibit anchorage-dependent and – independent growth of prostate cancer cells, and can enhance the apoptosis-inducing potential of TRAIL.

In order to examine the involvement of death receptor pathway on interactive effects of curcumin plus TRAIL, we used dominant negative FADD (DN-FADD). We have previously shown that DN-FADD blocked the interactions of TRAIL with histone deacetylase inhibitors, resveratrol, ionizing radiation or anticancer drugs [23,33,34]. As before, curcumin induced apoptosis in both PC-3/neo and LNCaP/neo cells (Fig. 1E and 1F). Curcumin enhanced the apoptosis-inducing potential of TRAIL in PC-3/neo cells, and sensitized TRAIL-resistant LNCaP/neo cells to undergo apoptosis. Transfection of DN-FADD had no effect on curcumin-induced apoptosis in both PC-3 and LNCaP cells, whereas it inhibited TRAIL-induced apoptosis in PC-3 cells. The ability of curcumin to enhance TRAIL-induced apoptosis in PC-3 cells, and its sensitization of TRAIL-resistant LNCaP cells was blocked by DN-FADD. These data suggest that death receptor pathway is involved in transducing effects of curcumin and TRAIL.

### Curcumin induces expression of death receptors

**Figure 2:**
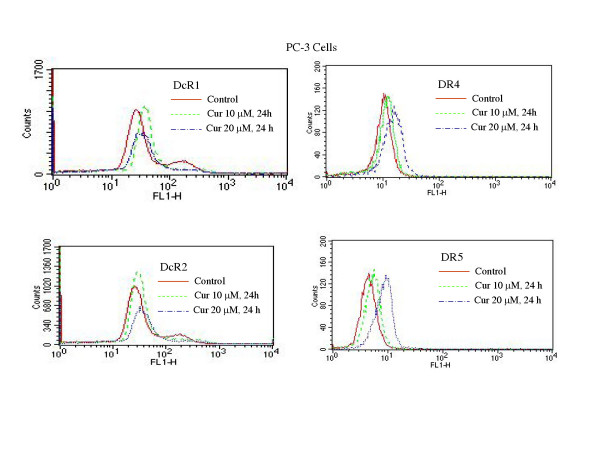
**Effects of curcumin on cell surface expression of death receptors**. PC-3 cells were treated with curcumin (0, 10 and 20 μM) for 24 h, and the expression of DcR1, DcR2, DR4, and DR5 was measured by flowcytometric analysis.

**Figure 3:**
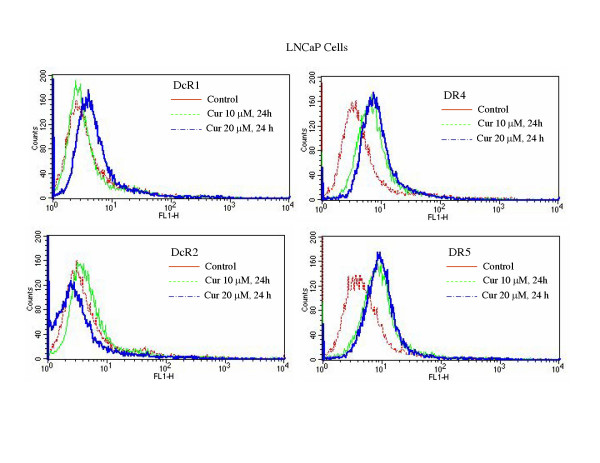
**Effects of curcumin on cell surface expression of death receptors**. LNCaP cells were treated with curcumin (0, 10 and 20 μM) for 24 h, and the expression of DcR1, DcR2, DR4, and DR5 was measured by flowcytometric analysis.

Activation of death receptors (TRAIL-R1/DR4 and TRAIL-R2/DR5) by TRAIL transmits caspase-dependent apoptosis signal [35]. We have recently shown that chemotherapeutic drugs, histone deacetylase inhibitors and γ-irradiation upregulate death receptors DR4 and/or DR5, and thus enhance the effectiveness of TRAIL [22,23,33,34,36,37]. Since curcumin enhances the apoptosis-inducing potential of TRAIL, we measured the expression of cell surface death receptors by flowcytometry (Fig. 2 and 3). Curcumin had no significant effect on the expression of DcR1 and DcR2 in PC-3 (Fig. 2) and LNCaP (Fig. 3) cells. By comparison, treatment of these cells with curcumin resulted in an increased expression of DR4 and DR5 in both cell lines. These data suggest that upregulation of death receptor DR4 and DR5 by curcumin may enhance the apoptosis-inducing potential of TRAIL in PC-3 and LNCaP cells.

### Regulation of Bcl-2 family members by curcumin

**Figure 4:**
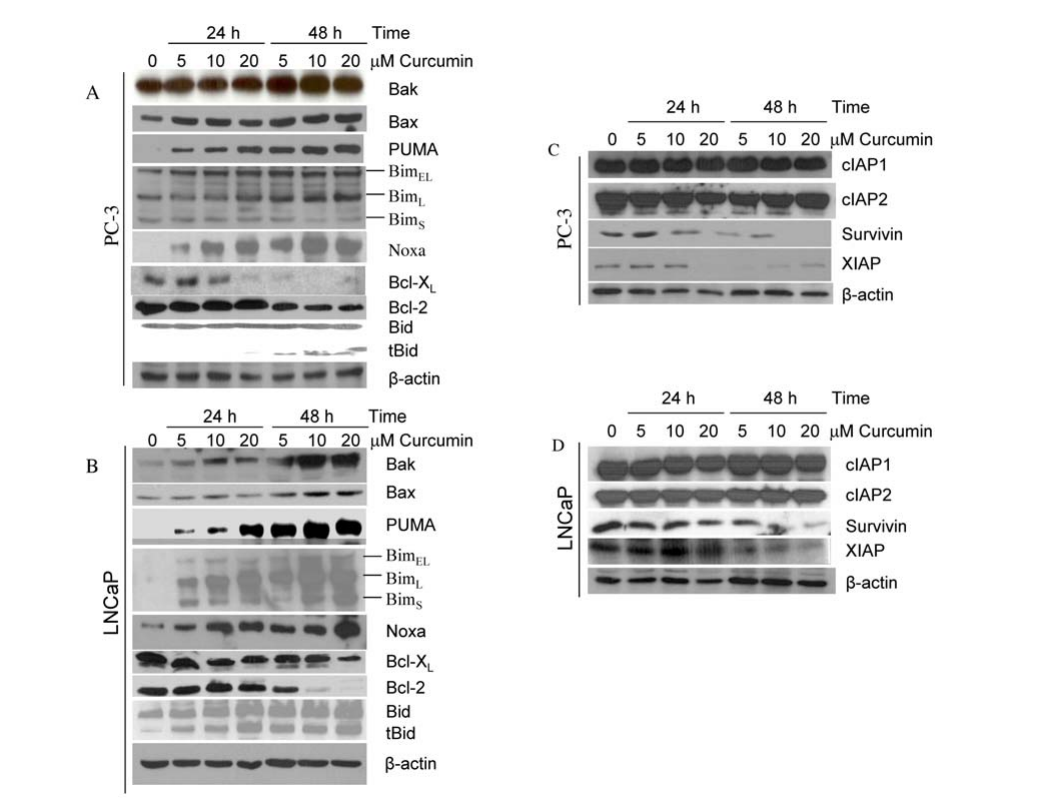
**Effects of curcumin on the expression of Bcl-2 family members, and inhibitors of apoptosis proteins (IAPs)**. (A and B), PC-3 and LNCaP cells were treated with or without curcumin (0, 5, 10 and 20 μM) for 24 or 48 h, and the expression of Bcl-2 family members (Bak, Bax, PUMA, Bim, Noxa, Bcl-X_L_, Bcl-2 and Bid) was measured by the Western blot analysis. β-actin was used as a loading control. (C and D), PC-3 and LNCaP cells were treated with or without curcumin (0, 5, 10 and 20 μM) for 24 or 48 h, and the expression of IAPs (cIAP1, cIAP2, survivin and XIAP) was measured by the Western blot analysis. β-actin was used as a loading control.

Bcl-2 family members regulate apoptosis induced by stress stimuli primarily at the level of mitochondria [34,38,39]. We, therefore, examined the effects of curcumin on the expression of Bcl-2 family members. The Western blot analysis showed that curcumin induced expression of pro-apoptotic proteins Bak, Bax, PUMA, Bim and Noxa, and inhibited expression of anti-apoptotic protein Bcl-X_L _and Bcl-2 in PC-3 cells (Fig. 4A). It is important to note that the expression of BimL isoform was significantly higher with all the doses of curcumin treatment at 48 h compared to control. The cleavage of Bcl-2 family member Bid to tBid (truncated Bid) is essential for linking death receptor pathway to mitochondrial pathway. We therefore measured whether curcumin treatment causes cleavage of Bid. Treatment of PC-3 cells with curcumin resulted in cleavage of Bid to tBid. Similarly, curcumin induced expression of Bak, Bax, PUMA, Bim and Noxa, and inhibited expression of Bcl-X_L _and Bcl-2 in LNCaP cells (Fig. 4B). Furthermore, Bid was cleaved by curcumin in LNCaP cells. These data suggest that Bcl-2 family members may regulate sensitivity of prostate cancer cells to curcumin and/or TRAIL.

### Inhibition of XIAP and survivin by curcumin

Inhibitors of apoptosis proteins (IAPs) play a major role in inhibiting caspase activation and apoptosis [40]. Since curcumin augments TRAIL-induced apoptosis by activation of caspases, we sought to examine the effects of curcumin on the expression of IAPs (XIAP, survivin, cIAP1 and cIAP2) in PC-3 and LNCaP cells (Fig. 4C and 4D). These cells were treated with curcumin for 48 h, and expressions of IAPs were measured by the Western blot analysis. Curcumin inhibited expression of survivin and XIAP, and had no effect on levels of cIAP1 and cIAP2 in both PC-3 and LNCaP cells. These data suggest that inhibition/cleavage of IAPs by curcumin may be one of the mechanisms regulating apoptosis.

### Interactive effects of curcumin and TRAIL on mitochondrial membrane potential

**Figure 5:**
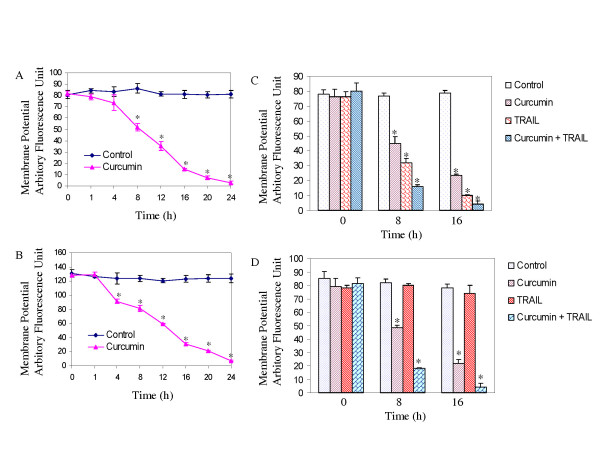
**Effects of curcumin on mitochondrial membrane potential**. (A and B), Curcumin induces drop in mitochondrial membrane potential (Δψm). PC-3 and LNCaP cells were treated with or without curcumin (20 μM) for 0–24 h. Cells were stained with JC1 dye, and Δψm was measured by fluorometer as per manufacturer's instructions. (C and D), Interactive effects of curcumin and TRAIL on Δψm. PC-3 and LNCaP cells were treated with or without curcumin (20 μM) in the presence or absence of TRAIL for 0, 8, or 16 h. Cells were stained with JC1 dye to measure Δψm. * = significantly different from respective control (P < 0.05).

During apoptosis, engagement of the mitochondrial pathway involves the permeabilization of the outer mitochondrial membrane (OMM), which leads to the release of proteins such as cytochrome c and Smac/DIABLO [41]. OMM permeabilization depends on activation, translocation and oligomerization of multidomain Bcl-2 family proteins such as Bax or Bak. We have shown that curcumin causes drop in mitochondrial membrane potential (ΔΨ_m_), and release of Smac/DIABLO, cytochrome c and Omi/HtrA2 from mitochondria to cytosol in prostate cancer cells [12]. Curcumin treatment of PC-3 and LNCaP cells caused a drop in ΔΨ_m _in a time-dependent manner (Fig. 5A and 5B). Treatment of these cells with curcumin, TRAIL or curcumin plus TRAIL had no effect on mitochondrial membrane potential at 0 h (Fig. 5C and 5D). In PC-3 cells, curcumin and TRAIL alone caused a ΔΨ_m _at both 8 and 16 h (Fig. 5C). The combination of curcumin and TRAIL had enhanced loss of ΔΨ_m _when compared with either agent alone. In LNCaP cells, although TRAIL alone was ineffective in reducing ΔΨ_m_, in combination with curcumin it caused significant loss of ΔΨ_m_(Fig. 5D). These data suggest that curcumin exerts antiproliferative effects at the level of mitochondria.

### Curcumin augments TRAIL-induced apoptosis through caspase activation

**Figure 6:**
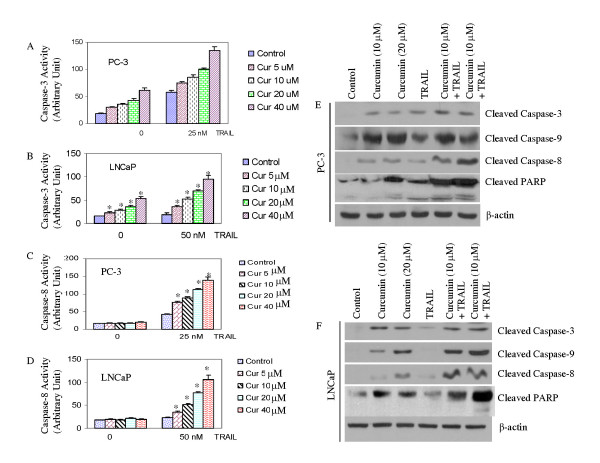
**Interactive effects of curcumin and TRAIL on caspase activation and PARP cleavage**. (A and B), PC-3 and LNCaP cells were treated with curcumin (0–40 μM), in the presence or absence of TRAIL, and caspase-3 activity was measured by fluorometric assay as per manufacturer's instructions. (C and D), PC-3 and LNCaP cells were treated with curcumin (0–40 μM), in the presence or absence of TRAIL, and caspase-8 activity was measured by fluorometric assay as per manufacturer's instructions. (E and F), PC-3 and LNCaP cells were treated with curcumin (0, 10 or 20 μM), in the presence or absence of TRAIL (25 nM for PC-3, and 50 nM for LNCaP), and the cleavage of caspase-3, caspase-9, caspase-8 and PARP was measured by the Western blot analysis. β-actin was used as a loading control.

The apoptotic death of cells requires proteolytic activation of caspases which are synthesized as latent proenzymes. Once activated, caspases cleave a wide range of molecules (e.g. PARP) that eventually result in the dismantlement of cells. Active caspases can be specifically inhibited by inhibitors of apoptosis (IAP). IAP antagonists such as Smac/DIABLO and Omi/HtrA2 compete with caspases for IAP-binding and consequently relieve caspase inhibition by IAPs and promote cell death. Since curcumin augments TRAIL-induced apoptosis, we sought to examine the activation/cleavage of caspase-9, -3 and -8, and PARP (Fig. 6). Curcumin induced caspase-3 activity in PC-3 and LNCaP cells (Fig. 6A and 6B). TRAIL induced caspase-3 activity in PC-3 cells, but had no effect in LNCaP cells. Curcumin has no effect on caspase-8 activity in PC-3 and LNCaP cells (Fig. 6C and 6D). However, TRAIL induced caspase-8 activity in PC-3 cells but not in LNCaP cells. Interestingly, the combination of curcumin and TRAIL resulted in more caspase-3 and caspase-8 activities in both cells lines (Fig. 6A–D).

PC-3 and LNCaP cells were treated with curcumin for 24 h followed by treatment with TRAIL for another 24 h, and the cleavage of caspase-3, caspase-9, caspase-8, and PARP was determined. Treatment of PC-3 cells with curcumin slightly increased caspase-3, caspase-9, caspase-8, and PARP cleavage (Fig. 6E). The combination of curcumin and TRAIL enhanced the cleavage of caspase-3, caspase-9, caspase-8 and PARP in PC-3 cells. In LNCaP cells, curcumin induced cleavage of capase-3, caspase-9, caspase-8 and PARP, whereas TRAIL had no effect on the cleavage of these proteins (Fig. 6F). Interestingly, pretreatment of curcumin with TRAIL resulted in cleavage of capase-3, caspase-9, caspase-8 and PARP. These data suggest that curcumin induces apoptosis through caspase activation. It is important to not that the combined treatments were more robust in the case of caspase-8 and PARP cleavage.

### Curcumin inhibits capillary tube formation by blocking ERK activity

**Figure 7:**
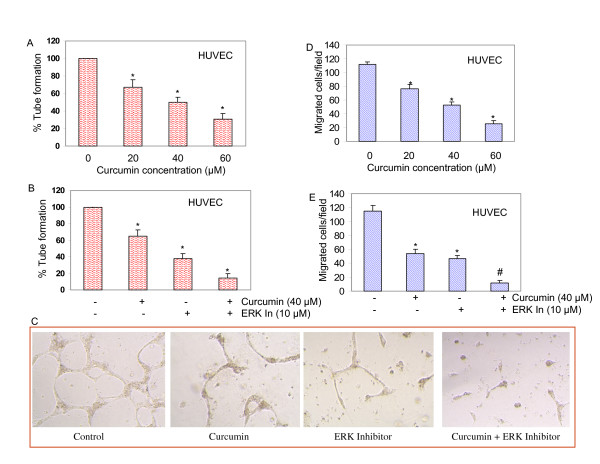
**Curcumin inhibits capillary tube formation and migration of HUVEC cells**. (A), HUVECs were seeded in 24-well plates containing matrigel, and treated with various concentrations of curcumin for 24 h. Capillary tubes were counted under a microscope. Data represent mean ± SD. * = significantly different from control, P < 0.05. (B), HUVECs were seeded in 24-well plates containing matrigel. Cells were pretreated with ERK inhibitor (10 μM) for 3 h, followed by treatment with curcumin (40 μM) for 24 h. Capillary tubes were counted under a microscope. Data represent mean ± SD. * = significantly different from control, P < 0.05. (C), Picture of capillary tube formation. HUVECs were treated as described in B. Pictures of capillary tubes were taken by a microscope. (D), HUVECs were treated with various concentrations of curcumin (20, 40 and 60 μM) or DMSO (control). Migration of HUVEC cells through the membrane was determined after 24 h of incubation at 37°C using Transwell Boyden chamber. Cells that had migrated to the lower chamber were fixed with 90% ethanol, stained with hematoxylin and eosin, quantified by counting the number of cells under a microscope. Data represent mean ± SD. * = significantly different from control, P < 0.05. (E), HUVECs were pretreated with ERK inhibitor (10 μM) for 3 h, followed by treatment with curcumin (40 μM) or DMSO (control) for 24 h at 37°C. Cells migrated to the lower chamber were fixed, stained and quantified.

Because curcumin treatment significantly decreased HUVEC viability, we sought to examine whether curcumin inhibited *in vitro *angiogenesis. We explored this possibility by determining the effect of curcumin treatment on capillary tube formation by HUVEC on growth factor-reduced matrigel, which is well-accepted technique to measure *in vitro *angiogenesis [42]. The data revealed that curcumin inhibited capillary tube formation in a dose-dependent manner (Fig. 7A). We next examined the involvement of ERK on capillary tube formation by using a specific inhibitor of ERK pathway. As shown in Fig. 7B and 7C, curcumin and ERK inhibitor alone inhibited capillary tube formation, and this effect of curcumin was further enhanced in the presence of ERK inhibitor. These results indicated that curcumin inhibition of capillary tube formation involved ERK MAP kinase.

### Curcumin inhibits migration of HUVEC cells through ERK inhibition

Next we determined the effect of curcumin treatment on invasion potential (migration) of HUVEC cells using a modified Boyden Chamber assay. In DMSO-treated controls, a large fraction of HUVEC migrated to the bottom face of the membrane (Fig. 7D and 7E). Treatment of chambers with curcumin resulted in inhibition of migration of HUVECs in a dose-dependent manner (Fig. 7D). Inhibition of ERK blocked migration of HUVEC cells, and the inhibitory effects of curcumin on cell migration were further enhanced in the presence of ERK inhibitor (Fig. 7E). These results indicated that the migration by HUVEC cells was inhibited by curcumin, and ERK is an important regulator of this process.

## Discussion

In the present study, we have shown that curcumin induces apoptosis in TRAIL-sensitive PC-3 cells, and sensitizes TRAIL-resistant LNCaP cells through activation of multiple signaling pathways. Curcumin-induced apoptosis engages mitochondria, which was evident by drop in mitochondrial membrane potential and activation of caspase-3 and caspase-9 in both androgen insensitive PC-3 and androgen-sensitive LNCaP cells. Curcumin induces expression of proapoptotic proteins (Bax, Bak, PUMA, Noxa and Bim), death receptors (TRAIL-R1/DR4 and TRAIL-R2/DR5), and inhibited expression of antiapoptotic proteins (Bcl-2 and Bcl-X_L_) and IAPs (XIAP and survivin). These proteins have been shown to regulate cell-intrinsic and/or cell-extrinsic pathways of apoptosis, and may be responsible for sensitization of TRAIL-resistant LNCaP cells.

Our recent studies have shown that curcumin downregulated the expression of Bcl-2, and Bcl-X_L _and upregulated the expression of p53, Bax, Bak, PUMA, Noxa, and Bim at mRNA and protein levels in prostate cancer cells [12]. We also showed that curcumin upregulated the expression, phosphorylation, and acetylation of p53 in androgen-sensitive LNCaP cells [12]. The ability of curcumin to regulate gene transcription was also evident as it caused acetylation of histone H3 and H4 in LNCaP cells [12]. Furthermore, treatment of LNCaP cells with curcumin resulted in translocation of Bax and p53 to mitochondria, production of reactive oxygen species, drop in mitochondrial membrane potential, release of mitochondrial proteins (cytochrome c, Smac/DIABLO and Omi/HtrA2), and activation of caspase-3 leading to apoptosis [12]. In another study, we have demonstrated that deletion of Bax and Bak genes completely inhibited curcumin-induced cytochrome c and Smac/DIABLO release in mouse embryonic fibroblasts [13]. The disruption of mitochondrial homeostasis by curcumin suggests that it can engage cell-intrinsic pathway of apoptosis.

We have shown that activation of death receptor pathway by TRAIL plays a major role in apoptosis [25,35]. The upregulation of death receptors by chemotherapeutic drugs, irradiation and chemopreventive agents have been shown to sensitize cancer cells to TRAIL treatment [23-25,33,34,36,37]. In the present study, we have demonstrated that curcumin enhances the therapeutic potential of TRAIL in PC-3 cells and sensitizes TRAIL-resistant LNCaP cells by upregulating DR4 and DR5, and regulating Bcl-2 family members and IAPs. Furthermore, curcumin potentiates the apoptotic effects of chemotherapeutic agents and cytokines through down-regulation of NFκB and NFκB-regulated gene products in bladder cancer [16], prostate cancer [43], and ovarian caner [21]. TNF-induced NFκB-regulated gene products involved in cellular proliferation (cyclooxygenase-2, cyclin D1, and c-myc), antiapoptosis [inhibitor of apoptosis protein (IAP)1, IAP2, X-chromosome-linked IAP (XIAP), Bcl-2, Bcl-X_L_, Bfl-1/A1, TNF receptor-associated factor 1, and cellular Fas-associated death domain protein-like interleukin-1beta-converting enzyme inhibitory protein-like inhibitory protein], and metastasis (vascular endothelial growth factor, matrix metalloproteinase-9, and intercellular adhesion molecule-1) were also down-regulated by curcumin [7]. These findings suggest that curcumin, in addition to activating cell-intrinsic pathway, can regulate cell-extrinsic (death receptor) pathway of apoptosis and may circumvent chemoresistance to conventional chemotherapeutic agents or TRAIL. We have also demonstrated that another chemopreventive agent resveratrol enhanced the apoptosis-inducing potential of TRAIL in PC-3 cells [44] and sensitized TRAIL-resistant LNCaP cells [45] by engaging both cell-intrinsic and cell-extrinsic pathways of apoptosis.

The serine/threonine kinase Akt/PKB has been considered an attractive target for cancer therapy and prevention [46]. Akt kinase plays critical roles in mammalian cell survival and is constitutively active in various cancers [12,46-48]. We have recently shown that curcumin upregulates the expression, phosphorylation (at serine 15) and acetylation of p53 [12]. Treatment of LNCaP cells with curcumin results in translocation of Bax and p53 to mitochondria, production of reactive oxygen species, drop in mitochondrial membrane potential, release of mitochondrial proteins (cytochrome c, Smac/DIABLO and Omi/HtrA2), activation of caspase-3 and induction of apoptosis [12]. Furthermore, curcumin inhibits expression of phosphatidylinositol-3 kinase (PI3K), and phosphorylation of Ser 473 Akt. Downregulation of Akt by inhibitors of PI3K (Wortmannin and LY294002) and Akt, or by dominant negative Akt increases curcumin-induced apoptosis, whereas transfection of constitutively active Akt attenuates this effect. Similarly, wild type phosphatase and tensin homolog deleted from chromosome 10 (PTEN) enhances curcumin-induced apoptosis. Furthermore, overexpression of constitutively active Akt inhibits curcumin-induced p53 translocation to mitochondria, and Smac release to cytoplasm, whereas inhibition of Akt by dominant negative Akt enhances curcumin-induced p53 translocation to mitochondria and Smac release [12]. These studies establish a role for Akt in modulating the direct action of p53 on the caspase-dependent mitochondrial death pathway and suggest that these important biological molecules interact at the level of the mitochondria to influence curcumin sensitivity. We have also demonstrated that curcumin causes acetylation of histone H3 and H4 in prostate cancer cells [12], suggesting histone modification may regulate gene transcription related to cell growth, differentiation and apoptosis.

Activation of the Raf-MEK-ERK signal transduction pathway in endothelial cells is required for angiogenesis [49]. Activating mutations in RAS and RAF result in inappropriate activation of downstream kinases, mitogen-activated protein kinase kinase (MEK) and extracellular signal-regulated kinase (ERK), and deregulated mitogenic and cell survival signaling [50]. The frequent derangement of these molecules in human cancers provides the rationale for their inhibition by pharmacological approaches. Our studies demonstrate that curcumin can inhibit capillary tube formation and endothelial cell migration, and these effects can be enhanced by MEK inhibitor. Curcumin has been shown to inhibit the expression genes involved in angiogenesis and metastasis (VEGF, ICAM-1, CD31, MMP-9) [7,9,10,15]. Thus, curcumin can inhibit tumor growth by inhibiting angiogenesis.

Curcumin has been shown to inhibit neoplastic initiation, promotion, and progression in several cancers. Curcumin inhibits tumor formation induced by ben(a)pyrene(BP), and 7,12-dimethylbenz(a)antracen (DMBA) in mouse model of gastric and skin tumors, respectively [51-53]. Furthermore, the administration of 4% curcumin in the diet of azoxymethane-treated mice reduced the number of colon tumors by 66% [54]. The clinical significance of curcumin *in vivo *is not yet clear. Data regarding the pharmacokinetics of curcumin suggest that there is a poor bioavailability in humans due to its rapid metabolism in the liver and intestinal wall, when taken orally [5,55-57]. Piperine has been shown to increase the bioavailability of curcumin by 2000% [58]. Curcumin significantly inhibited the cellular production of proinflammatory mediators such as TNFα and nitric oxide, and significantly inhibited the release of MCP-1 from adipocytes [59]. Curcumin can suppress obesity-induced inflammatory responses by suppressing adipose tissue macrophage accumulation or activation and inhibiting MCP-1 release from adipocytes, and thus it may have a potential to improve chronic inflammatory conditions in obesity. Curcumin is currently in clinical trials for the treatment of various cancers [57] and for Alzheimer's disease.

## Conclusion

Our study shows that curcumin enhances the apoptosis-inducing potential of TRAIL in PC-3 cells and sensitizes TRAIL-resistant LNCaP cells through multiple mechanisms. It induces death receptors, upregulates proapoptotic members of Bcl-2 family (Bax, Bak, Noxa, PUMA), inhibits antiapoptotic Bcl-2 proteins (Bcl-2 and Bcl-X_L_), IAPs (survivin, XIAP), and activates caspase-3, and caspase-9. The ability of curcumin to disrupt mitochondrial homeostasis may also be due to generation of reactive oxygen species as we have suggested previously [12]. Since curcumin activates both cell-intrinsic and – extrinsic pathways of apoptosis, it has enormous therapeutic potential. In addition to apoptosis, curcumin also inhibited capillary tube formation and invasion by blocking ERK. All these events will significantly contribute to the antiproliferative and antitumor activities of curcumin. Because curcumin has potent cytotoxic effects *in vitro*, especially in combination with TRAIL, studies to evaluate the efficacy of this combination in a suitable mouse model are warranted. Our studies strongly demonstrate that curcumin either alone or in combination with TRAIL can be used for prevention and/or treatment of prostate cancer.

## Methods

### Reagents

Antibodies against cIAP1, cIAP2, survivin, XIAP, Bcl-2, Bcl-X_L_, Bax, Bak, Bid, PUMA, Noxa, Bim, and β-actin were purchased from Santa Cruz Biotechnology Inc. (Santa Cruz, CA). Anti-caspae-3, anti-caspase-8, anti-caspase-9, and anti-PARP antibodies were purchased from BD Biosciences/Pharmingen (San Diego, CA). JC-1 was purchased from Invitrogen/Molecular Probes, Inc. (Eugene, OR). Enhanced chemiluminescence (ECL) Western blot detection reagents were from Amersham Life Sciences Inc. (Arlington Heights, IL). MEK1/2 inhibitor PD98059, and kits for Terminal Deoxynucleotidyl Transferase Biotin-dUTP Nick End Labeling (TUNEL), caspase-3 and caspase-8 assays were purchased from EMD Biosciences/Calbiochem (San Diego, CA). Antibodies against TRAIL-R1/DR4, TRAIL-R2/DR5, DcR1, and DcR2 for flowcytometry were purchased from R&D Systems, Inc. (Minneapolis, MN). TRAIL was purified as described elsewhere [60]. Curcumin was purchased from LKT Laboratories, Inc. (St. Paul, MN).

### Cell culture

Androgen-sensitive LNCaP and androgen-insensitive PC-3 cell lines from human prostate cancer were obtained from the American Type Culture Collection (Manassas, VA). Cultures were maintained in RPMI 1640 supplemented with 10% heat-inactivated fetal bovine serum (FBS) and 1% antibiotic-antimycotic (Invitrogen) at 37°C in a humidified atmosphere of 95% air and 5% CO_2_. Human umbilical vein endothelial cells (HUVECs) were purchased from Clonetics (Walkersville, MD) and maintained in endothelial cell growth factor medium-2 (EGM2 MV SingleQuots, Clonetics) supplemented with 5% FBS. The effect of curcumin on HUVEC viability was determined by trypan blue dye exclusion assay.

### XTT assay

Cell growth inhibition was analyzed by the spectrophotometric measurement of the mitochondrial dehydrogenase activity using 2,3-bis (2-methoxy-4-nitro-5-sulfophenyl)-5-[(phenylamino) carbonyl]-2H-tetrazo-lium hydroxide (XTT). XTT is converted to a colored formazan in the presence of metabolic activity (the primary mechanisms of XTT-to-formazan conversion are the mitochondrial succinoxidase and cytochrome P450 systems, as well as flavoprotein oxidases). Since the formazan product is water soluble, it is easily measured in cellular supernatants. Prostate cancer cells (1 × 10^4 ^in 200 μl culture medium per well) were seeded in 96-well plate (flat bottom), treated with or without drugs and incubated for various time points at 37°C and 5% CO_2_. Before the end of the experiment, 50 μl XTT labeling mixture (final concentration, 125 μM sodium XTT and 25 μM PMS) per well was added and plates were incubated for further 4 h at 37°C and 5% CO_2_. The spectrophotometric absorbance of the sample was measured using a microtitre plate (ELISA) reader. The wavelength to measure absorbance of the formazon product was 450 nm, and the reference wavelength was 650 nm.

### Colony formation assay

Prostate cancer **c**ells were seeded on soft agar at a density of 500 per well in a six-well plate containing 1 ml of RPMI/10% fetal bovine serum. Cells were then incubated at 37°C in a humidified atmosphere containing 5% CO_2_. Colony growth was assessed by the size and number of colonies after three weeks. Colonies exceeding the minimum diameter of 80 μm were counted.

### Measurement of apoptosis

Apoptosis was measured by the terminal deoxynucleotidyl transferase-mediated nick end-labeling method, which examines DNA strand breaks during apoptosis. Briefly, 1 × 10^5 ^cells were treated with curcumin and/or TRAIL at the indicated doses for various time points at 37°C. Thereafter, cells were washed with PBS, air-dried, fixed with 4% paraformaldehyde, and then permeabilized with 0.1% Triton X-100 in 0.1% sodium citrate. After washing, cells were incubated with reaction mixture for 60 minutes at 37°C. Stained cells were mounted and analyzed under a microscope. In some cases, the data were confirmed by staining cells with Hoechst 33248.

### Capillary tube formation assay

Capillary tube formation assays were performed as we described earlier [61]. In brief, matrigel (100 μl) was added to wells of a 96-well culture plate and allowed to polymerize for 1 h at 37°C. To examine the effects of curcumin on *in vitro *angiogenesis, subconfluent HUVECs were resuspended in complete medium and added to matrigel containing wells (1 × 10^4 ^cells/well), and exposed to various concentrations of curcumin, MEK1/2 inhibitor (PD098059) or DMSO (control). The plates were incubated at 37°C in a humidified atmosphere of 95% air and 5% CO_2 _[62]. Capillary tube formations (tubular structure formations) on 3-D Matrigel were visualized after 24 h under an inverted phase-contrast microscope (×200), and photomicrographs were documented by a SPOT digital camera (Sterling Heights, MI). Tube formation was defined as straight cellular extensions joining two cell masses or at branch points.

### *In vitro *cell migration (invasion) assay

Cell migration assays were performed as we described earlier [61]. In brief, migration of HUVEC cells was assessed using Transwell Boyden chamber (Corning, Acton, MA) containing a polycarbonated filter with a pore size of 8-μM. HUVECs (4 × 10^4 ^cells in 0.2 ml) cells in complete medium was mixed with desired concentration of curcumin, MEK1/2 inhibitor (PD098059) or DMSO (control), and the cell suspension was added to the upper chamber. The lower chamber contained 0.6 ml of complete medium with the same concentration of curcumin, ERK inhibitor or DMSO. Migration through the membrane was determined after 24 h of incubation at 37°C. Cells remaining on the topside of the transwell membrane were removed using a cotton swab. The membrane was washed with ice-cold PBS. Cells that had migrated to the underside were fixed with 90% ethanol and stained with hematoxylin and eosin. Cell migration was quantified by counting the number of cells per field.

### Assays for caspase-3 and caspase-8 activities

Cells (4 × 10^4 ^per well) were seeded in a 96-well plate with 200 μl culture medium. Approximately 16 h later, cells were treated with various doses of curcumin and/or TRAIL to induce apoptosis. Casapse-3 and caspase-8 activities were measured as per manufacturer's instructions (EMD Biosciences) with a fluorometer.

### Western blot analysis

Cell pellets were lysed in RIPA buffer containing 1 × protease inhibitor cocktail, and protein concentrations were determined using the Bradford assay (Bio-Rad, Philadelphia, PA). Cell lysates were electrophoresed in 12.5% SDS polyacrylamide gels and then transferred onto nitrocellulose membranes. After blotting in 5% nonfat dry milk in TBS, the membranes were incubated with primary antibodies at 1:1,000 dilution in TBS-Tween 20 overnight at 4°C, and then secondary antibodies conjugated with horseradish peroxidase at 1:5,000 dilution in TBS-Tween 20 for 1 hour at room temperature. Protein bands were visualized on X-ray film using an enhanced chemiluminescence system.

### Measurement of mitochondrial membrane potential (ΔΨm)

Mitochondrial energization was determined by retention of JC-1 dye (Molecular Probes Inc., Eugene) as we described earlier [22,63]. Briefly, drug treated cells (5 × 10^5^) were loaded with JC-1 dye (1 μg/ml) during the last 30 min of incubation at 37°C in a 5% CO_2 _incubator. Cells were washed in PBS twice. Fluorescence was monitored in a fluorometer using 570-nm excitation/595-nm emission for the J-aggregate of JC1 [64]. ΔΨ_m _was calculated as a ratio of the fluorescence of J-aggregate (aqueous phase) and monomer (membrane-bound) forms of JC1.

### Statistical analysis

The mean and SD were calculated for each experimental group. Differences between groups were analyzed by one or two way ANOVA using PRISM statistical analysis software (GrafPad Software, Inc., San Diego, CA). Significant differences among groups were calculated at P < 0.05.
